# Genetically Modified T Cells to Target Glioblastoma

**DOI:** 10.3389/fonc.2013.00322

**Published:** 2013-12-31

**Authors:** Simone Krebs, Tania G. Rodríguez-Cruz, Christopher DeRenzo, Stephen Gottschalk

**Affiliations:** ^1^Center for Cell and Gene Therapy, Texas Children’s Hospital, Houston Methodist Hospital, Baylor College of Medicine, Houston, TX, USA; ^2^Texas Children’s Cancer Center, Texas Children’s Hospital, Baylor College of Medicine, Houston, TX, USA; ^3^Department of Pediatrics, Baylor College of Medicine, Houston, TX, USA; ^4^Department of Pathology and Immunology, Baylor College of Medicine, Houston, TX, USA

**Keywords:** glioblastoma, immunotherapy, T-cell therapy, genetically modified T cells

## Abstract

Despite advances in surgical procedures, radiation, and chemotherapy the outcome for patients with glioblastoma (GBM) remains poor. While GBM cells express antigens that are potentially recognized by T cells, GBMs prevent the induction of GBM-specific immune responses by creating an immunosuppressive microenvironment. The advent of gene transfer has allowed the rapid generation of antigen-specific T cells as well as T cells with enhanced effector function. Here we review recent advances in the field of cell therapy with genetically modified T cells and how these advances might improve outcomes for patients with GBM in the future.

## Introduction

Glioblastoma (GBM) is the second most common, but most aggressive primary brain tumor (1). Despite aggressive, multimodal therapy consisting of surgery, radiation, and chemotherapy, the outcome of patients with GBM remains poor with 5-year survival rates of <10% (1–3). Therapeutic resistance to conventional therapies is most likely caused by several factors. First, GBMs are protected by the blood-brain barrier, resulting in low concentrations of therapeutic agents at tumor sites. Second, GBMs harbor multiple mutations in key oncogenic signaling pathways including RTK/RAS/PI3K, p53, and rb pathways (4). Third, glioma-initiating cells, which are critical for the malignant phenotype of GBMs are chemo- and radiation-resistant (5, 6). Lastly, GBMs create a hostile immunosuppressive tumor microenvironment, which prevents the induction of anti-GBM-specific immune responses (7).

Immunotherapy has the potential to improve outcomes for patients with GBM since it does not rely on the cytotoxic pathways of the aforementioned conventional therapies. The most widely pursued immunotherapy for GBM is vaccines (8–10). While vaccines are safe and have prolonged survival in comparison to historical controls, few complete remissions have been observed. Nevertheless, several vaccines are currently in randomized Phase III clinical trials including one vaccine that targets an EGFR splice variant (EGFRvIII) and another that consists of tumor lysate pulsed dendritic cells (DCs) (10, 11). Conceptually, the adoptive transfer of T cells has several advantages over vaccines. T cells can be expanded *ex vivo* outside the immunosuppressive tumor microenvironment, and T cells can be genetically manipulated to confer specificity and enhance their effector function (12, 13). While clinical experience with genetically modified T cells for GBM is limited, recent successes in patients with melanoma, neuroblastoma, and hematological malignancies have highlighted their potent antitumor activity (14–20). Here we will review gene transfer into T cells (Table 1) and how this technology is being adapted for the immunotherapy of GBM.

**Table 1:** **Genetic modification of T cells**.

Goal	Transgenes
Antigen specificity	αβ TCR, CAR
T-cell expansion & persistence	Co-stimulatory molecules, cytokines
T-cell homing to tumor site	Chemokine receptors
Counteracting immunosuppression	
TGFβ	Dominant-negative receptor
IL-4	Chimeric cytokine receptor
MDSC, Tregs	IL-12, IL-15
FAS ligand	shRNA to silence FAS ligand
Safety	HSV-*tk*, inducible caspase, CD20
Integration of T-cell therapy with conventional therapies	
TMZ resistance	MGMT
Steroid resistance	Zinc-finger nuclease to target steroid receptor

## Tumor-Associated Antigens Expressed in GBM

Glioblastomas express tumor-associated antigens (TAA) that are potential targets for immunotherapy including T-cell therapy (21, 22). TAA expressed in GBM can be classified into four categories based on their expression pattern: (i) antigens resulting from mutations, translocations, or splice variants (e.g., EGFRvIII) (23), (ii) antigens encoded by cancer-germ line genes [e.g., melanoma-associated antigen (MAGE), sarcoma antigen (SAGE), and synovial sarcoma X (SSX) families] (21, 22), (iii) antigens encoded by genes that are over expressed in GBMs [e.g., human epidermal growth factor receptor 2 (HER2), interleukin (IL)-13 receptor α2 (IL-13Rα2), erythropoietin-producing hepatocellular receptor A2 (EphA2)] (21, 24, 25), and (iv) viral antigens [e.g., pp65 and IE1 antigen of cytomegalovirus (CMV)] (26–28). Besides TAA expressed in malignant GBM cells, antigens expressed by vascular endothelial cells [e.g., vasculature endothelial growth factor receptor 2 (VEGFR2)] of the tumor vasculature or by other stromal cells are potential targets for T-cell therapy.

## Genetic Modifications to Render T Cells Specific for GBM

Two genetic strategies are widely used to generate tumor-specific T cells. One approach relies on modifying T cells with T-cell receptor (TCR) genes and the other on introducing genes encoding chimeric antigen receptors (CARs) into T cells.

### α/β TCR gene transfer

Conventional TCRs consist of two chains (α and β) that form heterodimers. TCRs recognize peptides, which are derived from proteins, in the context of major histocompatibility complex (MHC) molecules expressed on the cell surface. Isolating TCRs for adoptive T-cell therapy requires the generation of T-cell clones and subsequent isolation and cloning of the specific TCR α and β chains (29). Following isolation, α and β chain genes are cloned into viral vectors to introduce them into T cells (13). Initial studies highlighted that misspairing between endogenous α/β and transgenic α/β TCR chains is a common problem; however several approaches have been developed to overcome this limitation. For example, the introduction of disulfide bonds or use of murine sequences in the transgenic TCR genes results in preferential pairing of the introduced α/β TCR chains (30, 31). Silencing the expression of endogenous α/β TCR by shRNAs or zinc-finger nucleases are other attractive options (32, 33) that result in preferential pairing of the transgenic TCR.

α/β TCRs have been isolated for several TAA including CEA, GP100, MAGE-A3, MART1, and NY-ESO-1 (14, 34–37). While not tested in patients with GBM, some of these antigens are expressed in GBMs. The safety and efficacy of α/β TCR T-cell therapy has been evaluated in patients with melanoma, sarcoma, colon cancer, and multiple myeloma. One of the first studies in humans with α/β TCR T cells demonstrated that the infusion of autologous polyclonal T cells expressing a MART1-specific α/β TCR was safe and induced objective tumor responses in 2 out of 15 lymphodepleted patients with melanoma (34). To increase response rates, the same group infused T cells expressing high affinity MART1- and gp100-specific α/β TCRs. While response rates increased, several patients developed toxicities, including skin rash, uveitis, and/or hearing loss, that were not associated with antitumor responses (14). NY-ESO-1-specific α/β TCR T cells have also been evaluated in patients with synovial sarcoma, melanoma, and myeloma (37, 38), and encouraging antitumor responses have been observed without off-target side effects. In contrast, recognition of low levels of antigens on normal tissues by CEA-specific α/β TCR T cells has been observed in humans (36). Additionally, two adverse events have been reported in humans with affinity-matured TCRs, which recognized similar antigens (35, 39). Specifically, infusion of MAGE-A3-specific α/β TCR T cells caused both fatal neurotoxicity due to recognition of MAGE-A12, and fatal cardiac toxicities due to recognition of titin.

In conclusion, clinical studies with α/β TCR-modified T cells have not only demonstrated the potency of adoptively transferred T cells, but also some of their clinical limitations. Nevertheless, α/β TCR-modified T-cell therapy should be explored for patients with GBM, especially, since local delivery of such cells should reduce the risk of “non-CNS off target” effects.

### Chimeric antigen receptor gene transfer

#### Chimeric antigen receptor design

**Figure 1:**
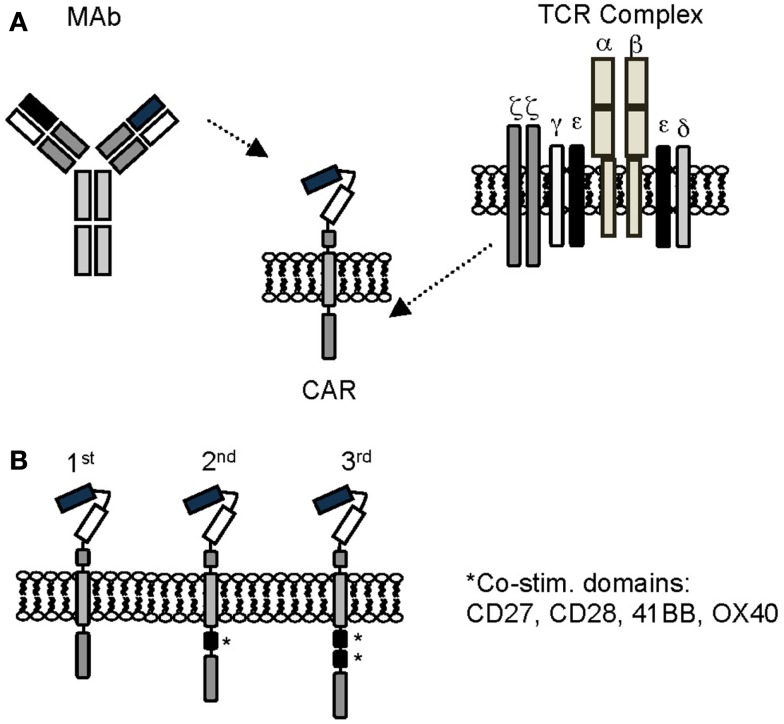
**Chimeric antigen receptors**. **(A)** Scheme of prototypic CAR. **(B)** First, second, and third generation CARs. See text for details.

Chimeric antigen receptors combine the antigen-binding property of monoclonal antibodies (MAbs) with the lytic capacity and self-renewal of T cells, and have several advantages over conventional T cells (Figure 1) (40, 41). CAR-expressing T cells recognize and kill tumor cells in an MHC unrestricted fashion, so that target cell recognition by CAR T cells is unaffected by some of the major mechanisms by which tumors avoid MHC-restricted T-cell recognition, such as downregulation of HLA class I molecules and defective antigen processing.

Chimeric antigen receptors consist of an ectodomain, commonly derived from a single chain variable fragment (scFv), a hinge, a transmembrane domain, and an endodomain with one (first generation), two (second generation), or three (third generation) signaling domains derived from CD3ζ and/or co-stimulatory molecules (41, 42). Besides scFvs, natural ligands of receptors or peptides have also been used as antigen recognition domains (43–46). While the potency of CARs correlates with the presence of co-stimulatory domains, the structural components of the CAR, like the hinge region or the transmembrane domain, and the level of CAR expression, contribute to overall CAR function (47, 48).

While the majority of CARs only recognize target antigens expressed on the cell surface, CARs can recognize carbohydrate and glycolipid antigens, increasing the pool of potential targets. Potential CAR targets expressed on the cell surface of GBMs include IL-13Rα2, HER2, EphA2, EGFRvIII, chondroitin sulfate proteoglycan 4 (CSPG4), and NKG2D ligands (Table 2) (21, 24, 25, 49, 50). Of these only EGFRvIII is solely expressed in GBM cells, raising concerns of “on target, off organ” side effects when EphA2, IL-13Rα2, or HER2 are targeted. Targeting multiple antigens might solve this conundrum. For example, investigators have engineered T cells expressing two CARs with complementary signaling domains to allow full T-cell activation only at tumor sites where both antigens are expressed (51–53), or have engineered a single CAR with two antigen-binding domains (54). Targeting multiple antigens is also advisable to offset immune escape, which has been observed for cancer vaccine as well as T-cell therapy studies in humans (10, 55, 56).

**Table 2:** **CAR T-cell therapy targets for GBM**.

GBM antigen	Studies	
	Animal^a^	Clinical
CSPG4	–	–
EGFRvIII	Yes (57, 58)	In progress
EphA2	Yes (59)	–
HER2	Yes (60)	In progress
IL-13Rα2	Yes (43, 61)	In progress (62)
NKG2D ligands	–	–

*^a^ GBM model*.

#### Preclinical studies with CAR T cells

Several investigators have evaluated the anti-GBM activity of CAR T cells in preclinical models (43, 57–61, 63–65). T cells expressing CARs specific for EphA2, EGFRvIII, IL-13Rα2, or HER2 recognized GBM cell lines or primary GBM samples in an antigen dependent manner as judged by cytokine production and cytolytic activity. CAR T cells also recognized and killed CD133-positive glioma-initiating cells, prevented neurosphere formation, and were able to destroy preformed neurospheres, demonstrating that radiation/chemotherapy-resistant glioma-initiating cells are sensitive to this immune-mediated killing mechanism (59, 60, 63, 64). *In vivo*, CAR T cells had potent antitumor activity in U87 and U373 xenograft models after local T-cell injection (43, 58, 59, 61, 65). In addition, investigators have demonstrated antitumor activity of CAR T cells using autologous GBM cells (60). Lastly, targeting two GBM antigens resulted in improved antitumor effects in one animal model (65). In summary, these studies demonstrate the potent anti-GBM activity of CAR T cells in preclinical models, warranting further active exploration. To date, CAR T-cell therapy for GBM has not been evaluated in immune-competent murine models. Immune-competent models will be critical to evaluate future combinatorial therapies in which adoptive T-cell transfer is combined with other agents to overcome the immunosuppressive tumor microenvironment.

While not tested in a GBM model, targeting the tumor vasculature with CARs is an attractive strategy to enhance the antitumor activity of CAR T cells (66, 67). Targeting the tumor vasculature with VEGFR2-specific CAR T cells in addition to tumor cells synergized in inducing tumor regression in several syngeneic, preclinical solid tumor models (68). In addition, transgenic expression of VEGFR2-specific CARs and IL-12 in T cells was sufficient to eradicate tumors, indicating that overcoming the inhibitory tumor microenvironment might potentiate effects of CAR T-cell therapies (see Section Engineering T-Cell Resistance to Immune Evasion Strategies Employed by GBMs) (69).

#### Clinical trials with T cells expressing CARs

So far clinical experience with CART-cell therapy for patients with GBM is limited. The safety and efficacy of intratumoral injection of T cells expressing a first generation IL-13Rα2-specific CAR (IL13-Rα2-CAR T cells) has been evaluated in one clinical study (62, 70). Infusion of IL13-Rα2-CAR T cells was well tolerated and associated with clinical benefit in several patients. In addition, two Phase I/II studies are currently in progress. In the first study the safety and efficacy of CMV-specific T cells expressing a second generation HER2-specific CAR is being evaluated in patients with recurrent GBM (NCT01109095). In the second study patients with recurrent GBM receive escalating doses of T cells expressing a third generation EGFRvIII-specific CAR after lymphodepleting chemotherapy (NCT01454596). Both studies are in progress, and clinical results should be available in the near future.

## Engineering T-Cell Resistance to Immune Evasion Strategies Employed by GBMs

Similar to other malignancies, GBMs create a hostile, immunosuppressive microenvironment (7, 71–73). They: (1) secrete immunosuppressive cytokines such as transforming growth factor β (TGF-β) or IL10, (2) attract immunosuppressive cells such regulatory T cells (Tregs) or myeloid derived suppressor cells (MDSCs), (3) inhibit DC maturation, (4) express molecules on the cell surface that suppress immune cells including FAS ligand (FAS-L) and PD-L1, and (5) create a metabolic environment (e.g., high lactate, low tryptophan) that is immunosuppressive.

Different approaches are being explored to overcome tumor induced immunosuppression. Such strategies include: (1) enhancing CAR T-cell expansion and persistence by providing co-stimulation and/or lymphodepletion, (2) transgenic expression of cytokines, (3) silencing negative regulators, and (4) expression of chimeric cytokine/chemokine receptors or signaling molecules.

### Increasing CAR T-cell expansion and persistence

Since T-cell expansion post antigen recognition requires the provision of co-stimulation, investigators have included signaling domains in CAR endodomains derived from co-stimulatory molecules including CD27, CD28, 4-1BB, and OX40. Several preclinical studies have highlighted the benefit of added co-stimulation (74–77); however only one study so far has done a “head to head comparison” of CD19-specific CARs with a ζ− or CD28. ζ-Domain in individual patients (78). While CD28 co-stimulation resulted in enhanced expansion of adoptively transferred T cells, the effect was limited.

Thus genetic modification alone might not be sufficient to allow for T-cell expansion *in vivo*. Dramatic T-cell expansion and long-term persistence post infusion of adoptively transferred T cells occurs in lymphodepleted patients post hematopoietic stem cell transplantation (79, 80). Investigators have therefore lymphodepleted patients outside the transplant setting prior to T-cell transfer. The extent of lymphodepletion correlated with antitumor effects (81), and therefore many investigators currently prefer to give lymphodepleting chemotherapy before adoptive transfer of conventional or CAR T cells. Another option to boost expansion of T cells *in vivo* is vaccination. For example expressing CARs in T cells that are specific for viruses allows for vaccination (e.g., influenza) (82) or stimulation by latently infected cells in humans (e.g., Epstein–Barr virus) (15). Besides co-stimulation, and the status of the lymphoid compartment, it is also apparent that subsets of T cells differ in their behavior *in vivo*. For example, expressing CARs in effector memory T cells can enhance T-cell persistence *in vivo* (83, 84). In addition, the presence of CD4-positive CAR T cells in the T-cell product has correlated with long-term T-cell persistence (16).

### Transgenic expression of cytokines

Chimeric antigen receptor T cells can be engineered to produce immunostimulatory cytokines. For example transgenic expression of IL-12 in CAR T cells reverses the immunosuppressive tumor environment by triggering apoptosis of inhibitory tumor-infiltrating macrophages, DCs, and MDSCs through a FAS-dependent pathway (85). While effective, there are safety concerns in regards to constitutive IL-12 expression. This obstacle can be overcome by using inducible promoters that are linked to the activation status of T cells, restricting IL-12 expression to tumor sites at which T cells are activated (86). Another attractive cytokine is IL-15. Transgenic expression of IL-15 (87, 88) renders T cells resistant to the inhibitory effects of Tregs through activation of the phosphoinositide 3-kinase (PI3K) (89), and improves CAR T-cell expansion and persistence *in vivo*.

### Silencing negative regulators

Silencing genes that render T cells susceptible to inhibitory signals in the tumor microenvironment has the potential to improve T-cell function. For example many tumor cells express FAS-L, and silencing FAS in T cells prevents FAS-induced apoptosis (90). Other options include silencing genes that encode inhibitory molecules expressed on the T-cell surface such as CTLA-4 or PD-1 (91).

### Expression of chimeric cytokine/chemokine receptors or signaling molecules

Transforming growth factor β is widely used by tumors as an immune evasion strategy (92), since it promotes tumor growth, limits effector T-cell function, and activates Tregs. These detrimental effects of TGF-β can be negated by modifying T cells to express a dominant-negative TGF-β receptor type II (DNR), which lacks most of the cytoplasmic kinase domain (93, 94). DNR expression interferes with TGF-β-signaling and restores T-cell effector function in the presence of TGF-β. The safety and efficacy of DNR-modified EBV-specific T cells is currently being evaluated in a Phase I/II clinical trial for patients with lymphoma (95), and if successful could be readily adapted to T-cell therapy for GBM.

T cells can also be engineered to convert inhibitory signals into stimulatory signals (96–98). For example, linking the extracellular domain of the TGF-β RII to the endodomain of toll-like receptor (TLR) four results in a chimeric receptor that not only renders T cells resistant to TGF-β, but also induces T-cell activation and expansion (98). Chimeric IL-4 receptors are another example of these “signal converters.” Many tumors secrete IL-4 to create a TH2-polarized environment, and two groups of investigators have shown that expression of chimeric IL-4 receptors, consisting of the ectodomain of the IL-4 receptor and the endodomain of the IL-7Rα or the IL-2Rβ chain, enable T cells to proliferate in the presence of IL-4 and retain their effector function including TH1-polarization (96, 97).

Another strategy to render T cells resistant to the inhibitory GBM environment is to express constitutively active signaling molecules. For example, expression of a constitutively active form of serine/threonine AKT (caAKT), which is a major component of the PI3K pathway, in T cells results in higher levels of NF-κB and elevated levels of anti-apoptotic genes such as Bcl2 conferring resistance to Tregs and TGFβ (99).

## Genetic Modification of T Cells to Improve Homing to Tumor Sites

While the intravenous administration of EBV-specific T cells resulted in the regression of CNS lymphoma (100), and the adoptive transfer of tumor-infiltrating lymphocytes (TILs) resulted in the regression of brain metastases (101), the homing of T cells to GBM sites might be suboptimal, similar to clinical experience with T-cell therapy for solid tumors. For example, in one clinical study with first generation folate receptor (FR)-α specific CAR T cells for patients with ovarian cancer, infused cells did not specifically home to tumor sites as judged by ^111^Indium scintigraphy, and no antitumor activity was observed (102). Since then, several investigators have shown in preclinical models that the expression of chemokine receptors in CAR T cells that recognize chemokines secreted by solid tumors can enhance T-cell homing. For example, transgenic expression of chemokine receptors CCR2b or CXCR2 in T cells enhances trafficking to CCL2- or CXCL1-secreting solid tumors including melanoma and neuroblastoma (103, 104). Thus, expressing chemokine receptors in T cells or adhesion molecules that potentially facilitate the infiltration of T cells into GBM tumors has the potential to enhance the antitumor efficacy of adoptively transferred T cells.

## Genetic Modification to Improve Safety of T-Cell Therapy

Potential toxicities can be divided into five categories: (1) toxicities due to genetic modification, (2) “on target organ” toxicities, (3) “on target, off organ” toxicities, (4) “off target, off organ” toxicities, and (5) systemic inflammatory syndromes. Toxicities due to genetic modification of T cells have not been observed in humans so far (105). An example of “on target organ” toxicity is the depletion of normal B cells after the infusion of CD19-speciifc CAR T cells for the treatment of B-cell malignancies (17). “On target, off organ” toxicity is exemplified by the liver toxicity observed after the infusion of carbonic anhydrase IX-specific CAR T cells to treat renal cell carcinoma (106). “Off target, off organ” toxicity is demonstrated by the recognition of MAGE-A12 in the brain or titin in the heart after the infusion of MAGE-A3-specific α/β TCR T cells for the treatment of melanoma, esophageal cancer, or myeloma (35, 39). Systemic inflammatory syndromes have been observed after CD19-specific CAR T-cell infusions for the immunotherapy of B-cell malignancies (17, 18, 107).

Genetic safety switches have been developed to selectively destroy genetically modified T cells once adverse events occur. The most widely used safety switch for T-cell therapy is the herpes simplex virus thymidine kinase (HSV-tk). HSV-tk phosphorylates acyclovir, valacyclovir, and ganciclovir to toxic nucleosides (108), and T cells transduced with the HSV-tk gene are killed in the presence of these medications. While clinical studies have demonstrated the effectiveness of this strategy, drawbacks to utilizing HSV-tk as a safety switch include the immunogenicity of HSV-tk, and that some patients require acyclovir, valacyclovir, or ganciclovir to treat herpetic diseases. Therefore, genetic safety switches using non-immunogenic human components have been developed, such as inducible caspase 9 (109, 110). With this strategy, once exposed to the dimerizer, genetically modified T cells rapidly undergo apoptosis. Another approach includes the transgenic expression of CD20, rendering T cells sensitive to the clinically approved CD20 MAb rituximab (111). While suicide gene switches can selectively kill infused cells, systemic inflammatory syndromes might be difficult to control since resident immune cells, activated by the infused T cells, most likely contribute. IL6 plays a critical role, and the infusion of the IL6 receptor MAb (tocilizumab) alone or in combinations with TNFα MAbs (infliximab) and steroids has proved to be effective (17, 18, 107).

While suicide switches are one strategy to prevent “on target, off organ” toxicities, T cells can also be engineered to only be fully activated if they encounter a unique “antigen address” at tumor sites. For example T cells expressing two CARs with different specificity of which one provides ζ-signaling and the other co-stimulation, will only be activated at tumor sites that express both antigens (51–53).

## Genetic Modification of T Cells to Facilitate Integration of Cell Therapy with Current Therapies

T cells are inherently sensitive to agents that are currently used for the treatment of GBMs including steroids and temozolomide (TMZ). Gene transfer can now be used to render T cells resistant to these agents. For example, disruption of the glucocorticoid receptor gene in T cells with zinc-finger nucleases results in T cells that function in the presence of steroids (112), and this strategy is currently being evaluated in a Phase I clinical trial (NCT01082926). TMZ resistance can be conferred by expressing *O*(6)-methylguanine-DNA-methyltransferase (MGMT) in T cells (113), potentially allowing the infusion of T cells while GBM patients receive TMZ.

## Conclusion

Preclinical studies and early clinical studies indicate that the genetic modification of T cells is a potent strategy to generate tumor-specific T cells with enhanced effector function. Not surprisingly, the greatest clinical success so far has been achieved for hematological malignancies targeting CD19. In order to develop effective CAR T-cell therapies for GBM several questions have to be addressed: which GBM antigen can be targeted without causing “on target/off cancer” side effects? How many antigens do we need to target to prevent immune escape? Do we have to target antigens expressed on non-malignant cells within the GBM microenvironment? Do we have to engineer T cells to enhance their homing to GBM sites and render them resistant to the immunosuppressive GBM environment? Do patients with GBM need to be lymphodepleted prior to T-cell transfer? Despite these unresolved issues, we believe that the results obtained so far with genetically modified T cells to target GBM are encouraging, warranting further active exploration. While genetically modified T cells are likely not the long awaited “GBM panacea,” integrating these cells into our current treatment armamentarium or combining them with other emerging targeted GBM therapies has the potential to improve outcomes for patients afflicted with this devastating cancer.

## Conflict of Interest Statement

The Center for Cell and Gene Therapy has a research collaboration with Celgene and Bluebird Bio. Simone Krebs, Christopher DeRenzo, and Stephen Gottschalk have patent applications in the field of T-cell and gene-modified T-cell therapy for cancer.
